# Regression-Based Normative Data for Independent and Cognitively Active Spanish Older Adults: Verbal Fluency Tests and Boston Naming Test

**DOI:** 10.3390/ijerph191811445

**Published:** 2022-09-11

**Authors:** Clara Iñesta, Javier Oltra-Cucarella, Esther Sitges-Maciá

**Affiliations:** 1 SABIEX, Universidad Miguel Hernández de Elche, Av. de la Universidad, 03207 Elche, Spain; 2 Department of Health Psychology, Miguel Hernández University of Elche, 03202 Elche, Spain

**Keywords:** cognitive impairment, dementia, executive function, language, memory disorders, neuropsychological tests, normative data

## Abstract

An increased cognitive reserve is associated with changes in the pattern of cognitive decline during aging. Thus, normative data adapted to the characteristics of the target population are needed to reduce the possibility of false diagnoses. The aim of this work was to develop normative data for the Phonemic Verbal Fluency test, the Semantic Verbal Fluency test and the Boston Naming Test (BNT). Method: Regression-based normative data were calculated from a sample of 118 non-depressed, cognitively active, independent community-dwelling adults aged 55 or older (64.4% women) from SABIEX (University for Seniors at the Universidad Miguel Hernández de Elche). Raw scores were regressed on age, sex, and education. Results: The effects of age and education varied across neuropsychological measures. No effect of sex was found in any of the tests assessed. Statistically significant differences were found in the proportion of low scores using SABIEX or population-based normative datasets. The level of agreement identifying individuals labeled as showing one or more low scores was only fair-to-good. Conclusions: Normative data obtained from the general population might not be sensitive to identify low scores in cognitively active older adults, increasing the risk of misdiagnoses. A friendly calculator is available for neuropsychological assessment.

## 1. Introduction

Increased life expectancy worldwide has resulted in a progressive growth in the proportion of people aged 65 or older and poses important challenges for the future. In Spain, 19.95% of the population is aged 65 or older and this percentage is expected to rise to more than 30% by 2050 [1]. As part of this worldwide aging, there has also been an increase in the rate of persons aged 85 and older, from 2.2% in 2010 to 3.3% in 2021.

Since age is the strongest risk factor for cognitive impairment [2], the aging of the population leads to an increased number of individuals at risk of cognitive impairment and dementia, particularly among the very old [3]. In 2020, the number of people living with dementia worldwide was estimated at 50 million [4]. It has been predicted that by 2040 the number of people affected will exceed 80 million [5]. In Europe, the number of people with Alzheimer’s Disease (AD), the most common form of dementia, is 7.7 million and it has been predicted that it will exceed 13 million by 2040 [6].

Currently, treatment options are still limited. Because of the absence of an effective disease modifying therapy, either pharmacological or non-pharmacological [7,8,9], dementia has become a global health challenge. Prevention and early intervention are the strategies that have been shown to be effective in delaying the appearance of symptoms of cognitive impairment and their progression to dementia. One of the primary targets in the field of dementias is to identify patients with mild cognitive impairment (MCI), due to its association with an increased risk of developing dementia [10]. From this perspective, neuropsychological assessment is essential to identify cognitive changes that exceed those expected as a part of normal aging and is a main part of the diagnosis of MCI and dementia. Thus, research has focused on the early identification of patients at risk of dementia, as the early detection of cognitive impairment through neuropsychological assessment would enable timely interventions.

The use of standardized neuropsychological tests depends on the availability of reliable normative data corrected for the influence on the performance of sociodemographic variables such as age, sex, or educational level. Since these adjusted normative data allow the interpretation of an individual’s performance, it is essential to have adequate normative data for the accurate interpretation of the results on the neuropsychological assessment [11].

Previous papers have reported the importance of using appropriate normative data, relevant to the population being assessed [12,13]. Normative data obtained in the general population might be less sensitive for identifying cognitive impairment among cognitively active older adults with higher performance levels independent of years of education. For example, people with low levels of education who are cognitively active are likely to be classified as having normal cognition if their performance is in the average range. However, being in the average range could mean low performance when compared to non or less active older adults, suggesting that cognitive impairment could be present. Since these individuals have higher levels of cognitive activity in comparison with people from the general population, their performance on cognitive tests is expected to be in the superior range. Previous works comparing the base rates of low scores using normative data obtained from the general population and normative data obtained with a sample of cognitively active older adults showed a lack of agreement in the identification of low scores using both normative datasets. Therefore, the accuracy in the performance’s interpretation might be maximized using population-specific normative data [12,13]. 

The number of older adults attending university courses in Spain is increasing in recent years, from 23,000 in 2005–2006 to 63,173 in 2019–2020 (www.aepumayores.org, accessed on 2 September 2022). Individuals attending university courses are motivated to feel more active, invest in personal development, gain new knowledge and social contacts [14,15], read more frequently, do more physical exercise, attend more cultural events, and participate more in social activities [16,17]. As cognitively older adults have higher cognitive reserve than community-dwelling individuals, specific normative data are essential to identify cognitive impairment. Our group developed in previous works normative data for the assessment of attention, processing speed, working memory [13], verbal and visual memory, and visuospatial perception [12] with a sample of cognitively active Spanish older adults who attend university courses. To complete a battery of neuropsychological tests covering all cognitive domains, the present study developed normative data on the assessment of semantic memory, executive functions, and language with a larger sample of cognitively active Spanish older adults who attend university courses. It is well documented that impairment in these cognitive functions is present in different neurodegenerative diseases [18,19,20,21]. Language impairment, related to different deficits and mechanisms underlying those deficits, is the predominant symptom of the three variants of Primary Progressive Aphasia (PPA) [22]. Deficits in verbal fluency (especially category fluency), naming, and semantic memory at an early stage are also well documented in AD, as well as the diagnostic utility of these tasks [23,24,25,26]. The identification and characterization of the language impairment pattern therefore plays an important role in the diagnosis of different types of dementias.

Verbal Fluency Tests (VFTs) are often used by neuropsychologists and researchers as these are brief, quick, and easy to administer. They are sensitive to many neurological conditions and are amongst the most widely used cognitive measures in dementia. The letters F, A, and S are the most used in English-speaking populations [11], whereas the letters P, M, and R have been proposed for Spanish-speakers based on the frequency of words [27]. There are two different types of VFTs: the letter and the semantic tasks. Although both category and letter fluency require efficiency in linguistics processes, processing speed, semantic memory, and impose significant demands on executive processes such as initiation, inhibition of inadequate responses, and ability to plan, organize, and monitor responses [28,29,30], they differ in terms of a stronger dependence on different cortical structures. Phonemic fluency is associated with a larger deficit on left frontal structures and semantic fluency is more dependent on left temporal structures [28,31,32,33]. Thus, the Phonetic Verbal Fluency test (PVF) is used as a measure of executive function while the Semantic Verbal Fluency test (SVF) is usually used as a measure of semantic memory. Some studies provided normative data for this test in Spain [34] and Latin America [35,36,37,38] with adults from the general population. 

Some studies with healthy controls and MCI and AD patients have reported the effectiveness of VFT to identify subtle cognitive impairment and to detect the conversion from normal cognition to MCI and from MCI to dementia [39,40,41]. It has been reported that, among other categories, the animal fluency test produces the best discrimination between healthy controls and MCI or AD patients [40,42,43] and of MCI to MCI-AD converters and appears to be the most useful VFT to predict conversion to probable dementia [40]. A longitudinal study reported that relatively poor semantic fluency performance among healthy adults may precede a decline in episodic memory as well as predict the progression to amnestic MCI [44]. It has also been reported that discrepancy scores between the PVF and SVF tests provide additional information of MCI patients who will progress to AD from those who will not, with a better prediction than individual fluency indices [45]. These findings emphasize the utility of VFTs in the early identification of AD risk.

The Boston Naming Test (BNT) [46] is a frequently used measure to assess language performance, specifically visual naming ability. Previous studies provided normative data for the BNT in the Spanish-speaking older population [47], and community-dwelling individuals over 50 in Spain within the Neuronorma Project [48] and in Latin America [49,50,51]. Some studies have reported the utility of the BNT in discriminating healthy adults from dementia patients [52,53]. Shortened versions of this test have been developed to improve the administration time and the efficiency of the assessment [54,55,56]. It has been reported that short versions are also valid to distinguish PPA and AD from healthy subjects [57]. Nevertheless, although abbreviated versions of the BNT seem to have similar psychometric properties, the ability to discriminate between healthy controls and MCI and between MCI and dementia patients seems to be stronger with the full version than with abbreviated forms [55]. 

These findings highlight the importance of including confrontation naming and verbal fluency tests in assessment batteries targeting the early identification of cognitive impairment. Thus, the aim of this study is to provide normative data for these three widely used neuropsychological tests. 

Developing normative data for these three tests for cognitively active older adults will allow a wider neuropsychological assessment and a more accurate interpretation of performance. Furthermore, it will improve the detection of cognitive impairment in this population, reducing the likelihood of diagnostic errors by using general population normative data. 

Since the cognitively active population is expected to have a higher cognitive reserve and higher performance independent of age and years of education [58,59], the hypothesis is that normative data obtained with a sample of cognitively active older adults will identify low scores with more certainty compared to normative data from the general population, as considering only the level of education in data from the general population might not capture higher levels of cognitive reserve. 

## 2. Materials and Methods

### 2.1. Participants

This was a cross-sectional observational study with cognitively healthy individuals living independently in the community. One-hundred and twenty participants were recruited from October 2019 to July 2022 from the University for Seniors (SABIEX) at the Universidad Miguel Hernández de Elche (Spain), an educational program for the promotion of active and healthy aging for people aged 55 years or older. SABIEX covers different topics such as history, literature, history of art, music, sociology, politics, botany, as well as different activities such as seminars, radio programs, writing a magazine, informatics, theater, and film workshops. 

Participants were included if they were (a) 55 years old or older, (b) cognitively normal (CN) without subjective cognitive complaints, and (c) independent in activities of daily living. Participants were classified as CN if they had (a) Mini-Mental State Examination [60] scores higher than 23, (b) Clinical Dementia Rating scale [61] scores equal to 0, and (c) Instrumental Activities of Daily Living [62] scores of 7 or higher. Potential participants were excluded if (a) they refused to participate in the neuropsychological assessment, and (b) if they had vision and/or hearing impairments that precluded the administration of cognitive tests. In order to assure a representative sample of the population over 54 years in Spain [63,64], participants were not excluded based on medical history (e.g., diabetes, high blood pressure, cancer, psychiatric disorders, metabolic disease). All participants were born and raised in Spain and had Spanish as their first language.

### 2.2. Procedure

Potential participants that voluntarily accepted to participate were assessed individually by a board-certified clinical neuropsychologist (JO-C) and trained undergraduate and Master’s or PhD degree students, and provided data regarding socio-demographics and personal and familial medical history. Prior to enrollment, an informed consent was obtained from all participants. The tests included in the neuropsychological battery have been previously reported [65], and included measures of attention, working memory, information processing speed, verbal and visual memory, visuospatial abilities, executive functioning, and language. This work was performed according to the Declaration of Helsinki and all participants provided an informed consent prior to enrollment. This project was approved by the UMH Ethics Committee (DPS.ESM.01.19; DPS.JOC.01.21). 

### 2.3. Materials

Subjective cognitive complaints were assessed with the Clinical Dementia Rating scale (CDR), general cognitive functioning with the Mini-Mental State Examination (MMSE) and depressive symptoms were assessed with the 30-item version of the Yesavage Geriatric Depression Scale [66] (GDS).

Phonemic and semantic fluency tasks were used to assess executive functioning and semantic memory, respectively. Language (naming) was assessed with the Boston Naming Test (BNT). These tests were administered in the following order:

#### 2.3.1. Phonemic Verbal Fluency (PVF)

In the PVF test, the examinee is requested to produce as many words as possible that begin with a specific letter for 60 s. In this study, the letters P, M, and R were used [34]. Participants were instructed to say any kind of word (nouns, verbs, adjectives, adverbs, pronouns) except proper nouns and to avoid augmentatives and diminutives of previously produced words. The outcome variable was the total number of correct words produced beginning with each letter. 

#### 2.3.2. Semantic Verbal Fluency (SVF)

The SVF test requires that the participant produce as many words as possible belonging to a specific category for 60 s [34]. In this study, the category “animals” was used. The outcome variable was the total number of correct (and unrepeated) animals. In both the PVF and SVF tests, higher scores indicate better performance. 

#### 2.3.3. Boston Naming Test (BNT)

The BNT is a visual-confrontation naming test that consists of black and white drawings of different objects arranged in order of increasing difficulty. The standard 60-item version of the BNT was used. Each item is presented consecutively, and the participant is requested to name each of the figures. If the participant does not know the name of the item or names it incorrectly, the examiner provides a standard semantic clue. If after that the participant still does not name the picture, a standard phonetic clue is provided [48]. The outcome variable was the total number of spontaneously named items and items named after the semantic clue. The maximum score is 60, with higher scores indicating a better performance.

### 2.4. Statistical Analyses

Statistical analyses have been used in previous works with the same sample and other neuropsychological tests [12,13]. A linear regression was built to predict each main outcome including age, sex, and education as predictors. Age and education were centered using the lowest value in the range of values (referred to as Age_Min_ and Education_Min_, respectively). Quadratic Age_Min_ and Education_Min_ were also included so as to analyze possible curvilinear relationships. The difference between the observed and predicted scores were standardized using the Standard Error of the Estimate (SEE), and z-scores equal to or lower than −1.28 were used to identify low scores. 

As with tests of attention, working memory, information processing speed [13], and verbal and visual memory [12], we compared both the rate of low scores and the agreement between SABIEX normative data and normative data obtained from the general population [34,48]. The rate of low scores was compared using the McNemar test (corrected for continuity) for related proportions [67], whereas the level of agreement between normative datasets (i.e., whether the same individuals are labeled as showing one or more low scores using both normative datasets) was compared using the Fleiss’ kappa [67] interrater correlation coefficient for categorical data. 

All statistical analyses were performed with the SPSS v.26 (IBM; Armonk, NY, USA). Statistical significance was set at 0.05.

## 3. Results

From a pool of 120 participants, two were not included because of MMSE scores < 24. The sample was composed of 118 participants (76 woman, 64.4%). Participants’ age ranged from 55 to 87 and years of education from 3 to 22 (not including University for Seniors). The descriptive statistics for demographic variables and MMSE, IADL, and GDS scores are provided in Table 1. Statistically significant differences were found between sexes in age (t (df = 116) = 2.89; *p* = 0.005), with men (M = 67.60; *SD* = 6.63) being older than woman (M = 64.08; *SD* = 6.15), but not in years of education (t (df = 116) = 1.095; *p* = 0.276), MMSE (t (df = 116) = −1.043; *p* = 0.299), IADL (t (df = 116) = 0.742; *p* = 0.46), or GDS (t (df = 101) = −0.859; *p* = 0.392). Most participants (62.7%) were married and were living with another person (75.4%). A total of 76 participants (64.4%) reported a history of medical illnesses (Table 2) and 50 (42.4%) were currently taking medication. A total of 32 participants (28.8%) reported having a familial history of dementia (e.g., parents, grandparents). The performance on neuropsychological tests is shown in Table 3. Statistically significant differences between sexes were only found on the PVF test letter “P”. Statistically significant differences were found between participants with or without medical history on the PVF test letter “P” (t (df = 116) = 2.00; *p* = 0.048), with participants reporting medical history (M = 14.43; SD = 4.55) showing worse performance than participants without it (M = 16.19; SD = 4.61). No statistically significant differences were found on performance between participants who were or were not taking medication (all *p*’s > 0.05). 

### 3.1. Calculation of Normative Data

The effects of age, sex, and education were different across the neuropsychological measures. The multiple linear regression models are presented in Table 4. Regression analyses showed that Age_Min_ was significantly associated with the BNT, SVF, and PVF letters “P” and “R”. Education_Min_ had significant effects on the BNT and PVF letters “P” and “R”. Education_Min_^2^ was associated with the PVF letter “M”. Sex had no effect on the neuropsychological tests included in this paper. Regarding the assumptions of regression analysis, the normal distribution of the residuals and multicollinearity (variance inflation factor [VIF] ≤ 10) was evaluated for all multiple linear regression models. VIF values in all models were below 10 and collinearity tolerance values did not exceed the value of 1 [68].

### 3.2. Comparing Normative Data Sets (NEURONORMA-SABIEX)

Using NEURONORMA normative data and taking a scaled score of six or lower as the cutoff for a low score, 17 participants (14.41%) had at least one or more low scores among the five measures. Using SABIEX normative data and a z-score *≤* −1.28 as the cutoff for a low score, 33 participants (27.97%) had at least one low score among these measures (Table S1). The McNemar test showed statistically significant differences in the proportion (*χ*^2^ (*N* = 118) = 12.500, *p =* 0.0004) of individuals with one or more low scores between normative data sets, and the Fleiss’s Kappa coefficient showed only a fair-to-good agreement identifying individuals labeled as showing one or more low scores using NEURONORMA and SABIEX normative data (k = 0.543, *p* = 0.000).

The number of low scores shown by fewer than 10% of the sample was two or more with both normative data: NEURONORMA (3.4%; SS < 6) and SABIEX (5.9%; *z* ≤ −1.28). MMSE scores were compared between individuals with and without low scores within normative data sets. Using SABIEX normative data, no statistically significant differences were found on the MMSE scores (t (df = 116) = 1.397; *p* = 0.165) between individuals classified as having one or more low scores (M = 28.24; *SD* = 1.54) and those showing no low scores (M = 28.67; *SD* = 1.47). Using NEURONORMA normative data, there were no statistically significant differences on the MMSE scores (t (df = 116) = 0.937; *p* = 0.351) between individuals with one or more low scores (M = 28.24; *SD* = 1.44) and those with no low scores (M = 28.60; *SD* = 1.51).

We provide a friendly calculator for researchers and clinicians that is available at: https://docs.google.com/spreadsheets/d/1p-RDT6F85EsXPxALV-l6R8Sq-E4qGEr0/edit#gid=665630718 (accessed on 2 september 2022).

## 4. Discussion

The aim of this work was to provide regression-based normative data for verbal fluency tests and the BNT for highly cognitively active Spanish older adults. These normative data complement a larger comprehensive neuropsychological battery assessing different cognitive domains [12,13]. As in previous works [35,36,50,69,70], quadratic age and education was included in the regression models to explore possible non-linear associations between these variables and performance in the tests.

### 4.1. Verbal Fluency Tests

There is inconsistency about the effect of sex on performance on verbal fluency tests in previous literature. Most studies reported a lack of association [34] or minimal effect size [36], whereas some studies reported sex differences only in semantic fluency and in specific categories but no others [34,38,70,71]. In our study, in agreement with most normative studies [34,35,36,38,69,72], no effect of sex was found in any of the verbal fluency tests.

#### 4.1.1. Semantic Verbal Fluency

Regarding the effects of age on performance, like previous studies with the adult population, our results show a negative linear relationship between age and SVF performance [34,36,69]. However, education had no significant effect on performance. This finding contrasts with previous studies in which education was associated with better performance on semantic fluency in the adult population and was a strong predictor of performance in verbal fluency for the animals category [34,35,36,69,72,73]. This result may support the idea that, independent of the level of education achieved, cognitively active lifestyles during adulthood have a higher influence creating cognitive reserve [59,74] and a greater impact on performance on cognitive tests [58,74]. 

#### 4.1.2. Phonetic Verbal Fluency 

In line with previous studies, age and education were significantly associated with PVF letters “P” and “R” [34,35,69]. Performance decreased with age and increased with education. Nevertheless, age did not have influence on the letter “M” and the effect of education on this letter was non-linear. The lack of effect of age as well as the curvilinear association with education was unexpected, as this letter should be influenced in a similar way to the other two letters by important independent variables such as age and education. It is unclear what caused this difference in the effect of age and education on this letter. These results are, however, in line with some studies in which quadratic education partially explained the variance of PVF tests [37,69], but in these cases, this may be at least partly explained by the wider age range of their samples (18–89).

### 4.2. Boston Naming Test

Regarding the effect of sex, the majority of studies report no significant differences between men and women or significant differences but with minimal effect size [50,72,75,76,77]. Our findings support a lack of association between this variable and performance on the BNT. However, some studies have reported a clear male advantage [47]. Hall et al. [78] also found significant gender differences, with men outperforming women in healthy controls as well as in AD groups. Regarding the effects of age and education, our findings are consistent with most previous studies reporting a significant effect on performance of these variables, with an increase in BNT scores with a higher level of education and a decrease with advancing age [34,38,50,72,76]. Our results show that both variables were associated with performance and were statistically significant to predict the total score. A relevant finding is the fact that in most of these studies with the adult population, the level of education was the best predictor of the test scores, accounting for the greatest proportion of variance in the final model. In the present work, education contributed little to its prediction beyond the contribution of age, with a small effect size. This is of interest in a sample of cognitively active adults. As reported in previous papers with this sample [12,13], this might show that education becomes less relevant when individuals are highly cognitively active during adulthood and that cognitive activities through a lifespan have a higher influence in determining cognitive reserve [58,59,79]. 

Additionally, the rate of low scores and the agreement between our normative data and normative data obtained from the general population was compared. The results showed statistically significant differences in the proportion of individuals with one or more low scores between both SABIEX normative data and NEURONORMA normative data and only a fair-to-good agreement in identifying low scores between both normative datasets. As in previous works with this population [12,13], the discrepancy found in the classification suggests that using normative data obtained in the general population might be inappropriate for the neuropsychological assessment of highly cognitively active older adults, with an increase in the number of misdiagnoses of cognitive impairment by erroneously identifying low scores. 

Regarding the clinical applicability, one strength of this work is that these norms were obtained as part of a larger battery of neuropsychological tests that will facilitate the comparisons of performance across different cognitive domains and therefore will improve the diagnosis of cognitive impairment and dementia in the cognitively active population.

### 4.3. Limitations

This work is subject to certain limitations. First, participants were recruited from university courses for seniors at the Universidad Miguel Hernández de Elche and the findings are not necessarily representative of all the adult population attending university programs for seniors (UPS). Furthermore, although participation in UPS has been related to a greater engagement in a variety of active practices [16,17], it would be recommendable to analyze whether these normative data are also appropriate to accurately interpret the performance of active older adults engaged in different cognitively stimulating activities (e.g., active leisure activities or volunteering, reading, and writing), which also positively impact cognitive functioning in older age [74,80,81]. Second, these normative data are limited for use with people between 55 and 87 years. Increased variability in cognitive function and performance in advancing age has been reported [82]. This supports the use of appropriate normative data sets for the oldest-old population. 

A further limitation is that these normative data were obtained in the Spanish population. Their use with other Spanish-speaking populations should be interpreted with caution, as the effect of different cultural backgrounds in performance on neuropsychological tests is well documented [11]. Previous works on normative data for Spanish-speaking countries have shown that individuals from different countries perform differently on the same neuropsychological test, making normative data from one country useless for individuals from another country [36,37,50].

Regarding the SVF test, our work focuses only on the category “animals”, which may be viewed as a limitation. The use of combined categories (e.g., fruits, vegetables, and professions) might be more reliable than a single category. Nevertheless, “animals” is the most frequently used category in clinical practice. Furthermore, it has been reported that this category produces the best discrimination between healthy controls and MCI patients and appears to be the most useful VFT to predict conversion to probable dementia [40,42].

Regarding the BNT, the 60-item version might be difficult to apply in terms of administration time in clinical assessment and it might be difficult to complete for severely impaired persons. Although multiple 12-, 15-, and 30-item short versions of the BNT [54,56,57,83,84,85,86] have been validated, show a high correlation with the original version, and might be useful in daily clinical practice, it has been reported that the full version has a stronger ability to discriminate between healthy controls and MCI and between MCI and dementia patients [55].

## 5. Conclusions

The normative data reported in the present work and previous papers [12,13] should be especially useful for clinicians and researchers to accurately interpret the performance of older adults who continue to lead a highly active life during aging, identifying low scores and impaired performance more accurately than with tests standardized in the general population and therefore reducing diagnostic errors in the cognitively active adult population.

## Figures and Tables

**Table 1:** Demographic statistics and performance on MMSE, IADL, and GDS.

Variable	*M*	*SD*	Range
Age	65.33	6.52	55–87
Education	11.69	3.48	3–22
MMSE	28.55	1.50	24–30
IADL	7.99	0.09	7–8
GDS	4.41	3.39	0–18

M: mean, SD: standard deviation, MMSE: Mini-Mental State Examination, IADL: Instrumental Activities of Daily Living, GDS: Geriatric Depression Scale.

**Table 2:** Number (and %) of participants with medical history (*n* = 118).

	*n*	%
Anxiety	19	16.1
Depression	9	7.6
Epilepsy	2	1.7
Stroke	4	3.4
CVD	14	11.9
Hypo/hyperthyroidism	8	6.8
Cancer	11	9.3
DM	4	3.4
HBP	16	13.6
TBI	3	2.5
COPD	1	0.8
RA	2	1.7
Others	19	16.1

CVD: cardiovascular disease, DM: diabetes mellitus, HBP: high blood pressure, TBI: traumatic brain injury, COPD: chronic obstructive pulmonary disease, RA: Rheumatoid arthritis, Others: hypercholesterolemia, osteopenia, chronic venous insufficiency, dyslipidemia, dyspepsia, cholecystectomy, autoimmune hypoglycemia, COVID-19, asthma, migraine, spina bifida occulta, and bipolar disorder.

**Table 3:** Raw scores on neuropsychological tests.

Neuropsychological Measures	*M*	*SD*	Range
PVF “P” (*n* = 117)	15.06	4.63	5–27
PVF “M” (*n* = 118)	13.64	4.14	5–23
PVF “R” (*n* = 118)	13.39	4.24	3–25
SVF (*n* = 118)	20.34	5.42	10–38
BNT (*n* = 118)	55.05	3.27	41–60

M: mean, SD: standard deviation, PVF: Phonetic Verbal Fluency, SVF: Semantic Verbal Fluency, BNT: Boston Naming Test.

**Table 4:** Multiple linear regression models.

		β	*SE* (β)	95% CI	p_coeff_	R^2^_Adjusted_
PVF “P”	Intercept	13.858	1.223	11.43–16.28	<0.001	
	Education_Min_	0.356	0.117	0.12–0.59	0.003	
	Age_Min_	−0.184	0.062	−0.31–−0.06	0.004	0.110
PVF “M”	Intercept	12.234	0.612	11.02–13.45	<0.001	
	Education_Min_^2^	0.016	0.006	0.005–0.027	0.005	0.058
PVF “R”	Intercept	12.347	1.124	10.12–14.57	<0.001	
	Education_Min_^2^	0.298	0.108	0.08–0.51	0.007	
	Age_Min_	−0.150	0.058	0.26–0.03	0.011	0.086
SVF	Intercept	23.414	0.878	21.68–25.15	<0.001	
	Age_Min_	−0.298	0.072	−0.44–−0.15	<0.001	0.121
BNT	Intercept	54.675	0.879	52.92–56.40	<0.001	
	Age_Min_	−0.110	0.045	−0.20–−0.02	0.017	
	Education_Min_	0.176	0.085	0.008–0.34	0.040	0.058

PVF: Phonetic Verbal Fluency, SVF: Semantic Verbal Fluency, BNT: Boston Naming Test, β: regression coefficient, *SE* (β): standard error of β.

## Data Availability

Data are available at request from the corresponding author.

## Supplementary Materials

The following supporting information can be downloaded at: https://www.mdpi.com/article/10.3390/ijerph191811445/s1, Table S1: Comparing number of low scores between normative data sets (NEURONORMA-SABIEX).
