# Supplementary material for: Regression-Based Normative Data for Independent and Cognitively Active Spanish Older Adults: Verbal Fluency Tests and Boston Naming Test
Source: Int J Environ Res Public Health. 2022 Sep 11;19(18):11445. doi: 10.3390/ijerph191811445 (PMC9517509; doi:10.3390/ijerph191811445)
Supplement: Supplementary file 1 [file ijerph-19-11445-s001.zip › ijerph-1878128-supplementary.pdf]

Supplementary material

Table S1. Comparing number of low scores between normative data sets (NEURONORMA-SABIEX).

		SABIEX		
		0	1+	Total
NEURONORMA	0	84	17	101
	1+	1	16	17
Total		85	33	118
